# Identification of Potential Auxin-Responsive Small Signaling Peptides through a Peptidomics Approach in *Arabidopsis thaliana*

**DOI:** 10.3390/molecules24173146

**Published:** 2019-08-29

**Authors:** Weigui Luo, Yuan Xiao, Qiwen Liang, Yi Su, Langtao Xiao

**Affiliations:** 1 Hunan Provincial Key Laboratory of Phytohormones and Growth Development, Hunan Agricultural University, Changsha 410128, China; 2 School of Life Sciences, Tsinghua University, Beijing 100084, China

**Keywords:** small signaling peptides, plant protoplast, peptidomics, auxin

## Abstract

Small signaling peptides (SSPs) are a class of short peptides playing critical roles in plant growth and development. SSPs are also involved in the phytohormone signaling pathway. However, identification of mature SSPs is still a technical challenge because of their extremely low concentrations in plant tissue and complicated interference by many other metabolites. Here, we report an optimized protocol to extract SSPs based on protoplast extraction and to analyze SSPs based on tandem mass spectrometry peptidomics. Using plant protoplasts as the material, soluble peptides were directly extracted into phosphate buffer. The interference of non-signaling peptides was significantly decreased. Moreover, we applied the protocol to identify potential SSPs in auxin treated wild type and auxin biosynthesis defective mutant *yuc2yuc6*. Over 100 potential SSPs showed a response to auxin in *Arabidopsis thaliana*.

## 1. Introduction

Small signaling peptides (SSPs) are a class of short peptides acting as signal molecules mediating cell-to-cell communication, regulation in plant growth and development, and plant response to the changing environment [1]. Similar to the conventional phytohormones, SSPs also have physiological functions through perception by their specific receptors via certain signal transduction pathways. Nowadays, it is gradually accepted that SSPs are a new class of phytohormones [2]. Most functional SSPs generally appear as mature peptides with less than 20 amino acid residues, produced by selective action of peptidases on a larger precursor or by degrading from the proteolytic enzymes [3]. SSPs can function locally through intracellular signaling or be delivered to the extracellular spaces. SSPs can be structurally categorized into two groups: secreted peptides and non-secreted peptides. Most of the previously identified SSPs are secreted peptides, which mainly consist of two major types—posttranslational modified (PTM) peptides and cysteine-rich peptides without posttranslational modification [4]. A few SSPs are non-secreted exceptions, such as *Arabidopsis* peptide 1 (Pep1) [5,6], early nodulin 40 (ENOD40) [7], ROTUNDIFOLIA (ROT/DVL) [8,9], and POLARIS (PLS) [10]. 

The first plant small signaling peptide named systemin was described in tomato [11]. Subsequently, series of signaling peptides were predicted and identified in plants. According to bioinformatic prediction, there might be more than 1200 SSPs in plants [12], but only a small portion of them have been systematically studied [1,13]. Up to date, dozens of SSP families have been identified and diverse roles of plant SSPs have been verified [1]. In short, known SSPs are mainly involved in defense response [14], cell expansion [15], embryogenesis [16], reproduction [17,18,19], root system architecture [20,21], vascular formation [22,23], stomatal development [24], and organ abscission [25]. However, current knowledge about plant SSPs is very limited and a large number of unknown SSPs need to be identified.

The reason why SSPs had been historically overshadowed was partly because of technical limitations. The challenge of SSP identification lies not only in their small molecular sizes, but also in their extremely low concentrations in plants [26]. Early identified SSPs, such as systemin and phytosulfokine (PSK), were discovered through biochemical assays [27]. Subsequently, genetics screening was introduced to the identification of CLAVATA 3 (CLV3) [28,29] and the CLV3/endosperm surrounding region (CLE) [23]. Recently, a proteomics approach based on tandem mass spectrometry (MS/MS) was employed to identify SSPs [30,31]. However, it is still hard to accurately distinguish SSPs from various non-signaling peptides in the complex tissue extract. As a result, a large number of false positive hits would be generated in the mass spectrogram. Protein and peptide databases and algorithm tools greatly facilitated the detection of peptides in plants [12,32]. The in silico prediction based on the structural features benefited the discovery of many reported plant peptides [33,34], but the mature forms of peptides cannot be identified by in silico approaches [35]. Therefore, in order to identify plant SSPs more efficiently, the technique for plant SSP identification still needs to be improved. Combination of bioinformatics and MS/MS methods are helpful for the technical progress of SSP analysis [14]. Recently, peptidomics, an omics approach based on tandem spectrometry, becomes a promising method to identify potential SSPs [36].

As naturally occurring signaling molecules, SSPs revealed close interaction with phytohormones in various developmental processes. For instance, the tomato Leu-rich repeat receptor kinase BRASSINOSTEROID INSENSITIVE1 (BRI1) is involved in the perception of both systemin and brassinosteroids (BRs) [37]. Ethylene perception is required for normal systemin responses in roots [37]. CLAVATA3 affects abscisic acid biosynthesis and stomatal control of transpiration in leaves [38]. CLE and cytokinin signaling are highly intertwined developmental regulators with antagonistic functions in shoots and synergistic functions in roots [39]. Auxin is one of the most critical signal molecules in the regulation of plant growth and development. Several SSPs were reported to interact with auxin. The POLARIS peptide in *Arabidopsis* regulates auxin transport and root growth via ethylene signaling [10]. A secreted peptide EPIDERMAL PATTERNING FACTOR-LIKE (EPFL) and its receptors ERECTA (ER) shape the auxin response pattern and leaf margin morphogenesis [40]. Small secretory peptide GOLVEN (GLV) modulated the distribution of auxin [41,42], while PSK initiates auxin-dependent immunity through cytosolic Ca^2+^ signaling in tomato [43]. Nevertheless, a lot of auxin-responsive SSPs need to be identified to further reveal the interactions between SSPs and auxin. The peptidomics approach provides a powerful tool in new SSP identification. In order to discover more unknown SSPs in *Arabidopsis* through the peptidomics approach, we optimized the peptide extraction condition based on protoplasts by using the auxin synthetic defective mutant *yuc2yuc6* [44,45] as the material. By analyzing the abundance of peptides through TOF tandem mass spectrometry, auxin-responsive SSP candidates were identified from the omics point of view. 

## 2. Results and Discussion

### 2.1. Optimization of Peptide Extraction

#### 2.1.1. Peptides Extracted by Different Lysate Buffer

**Figure 1:**
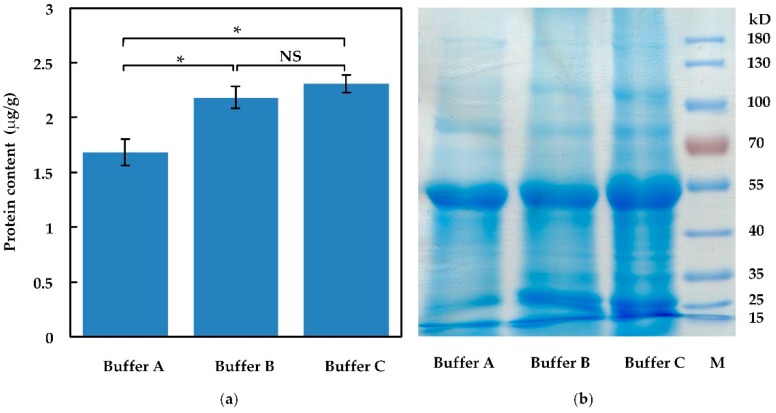
Total-protein (including peptides) extracted in different lysate buffers. (**a**) Total-protein yields extracted in different lysate buffers; (**b**) total-protein was visualized in SDS-PAGE. Asterisk (*) represents significant difference (*P* < 0.05). NS represents no significant difference. Error bar represents the mean ± standard deviation (*n* = 3).

As signaling molecules, SSPs play important roles at extremely low concentrations in plants. To improve the yield of SSP extraction, three different buffers were tested in our experiments. Aerial tissues of wild type *Arabidopsis* were collected and frozen in liquid nitrogen immediately. Frozen plant tissues were thoroughly ground into powder and the tissue powder was added into different lysate buffers with the same volume to extract total-protein. The total-protein (including peptides) contents were determined by Bradford assay, as shown in Figure 1a, and then stained by Coomassie brilliant blue in SDS-PAGE gel, as shown in Figure 1b. Total-protein yields were 1.68, 2.18, and 2.31 microgram protein per gram in plant tissues, respectively, extracted in buffer A, B, and C. The result indicated that buffer C (7 M urea, 2 M thiourea, 3.5 mM SDS, 0.01 M Tris, 2 mM EDTA, 10 mM DTT) is a preferable lysate to harvest total-protein (including peptides). Stained by Coomassie brilliant blue, more protein bands under 55 kD were visualized in the buffer C sample. Among the tested buffers, buffer C is more suitable for protein extraction.

#### 2.1.2. Peptides Extracted by Ultrasonic and Non-Ultrasonic Methods

**Figure 2:**
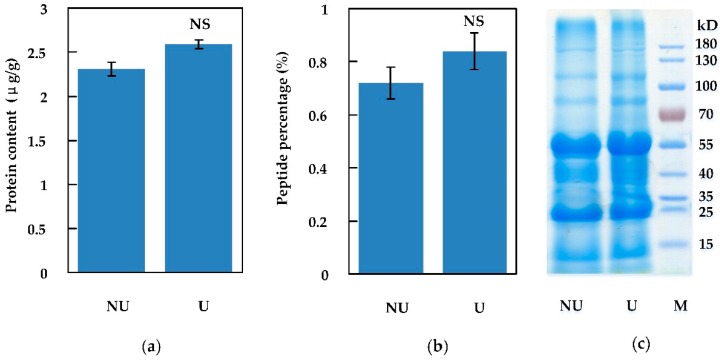
Peptides extracted by ultrafiltration and non-ultrafiltration. (**a**) Total-protein (including peptides) yield extracted by non-ultrafiltration (NU) and ultrafiltration (U); (**b**) peptide content by non-ultrafiltration (NU) and ultrafiltration (U); (**c**) total-protein (including peptides) visualized in SDS-PAGE. NS represents no significant difference. Error bar represents the mean ± standard deviation (n = 3).

In order to improve the peptide extraction yield through cell fragmentation, ultrasonication was applied. We compared the extraction efficiency between ultrasonic and non-ultrasonic methods. Total-protein (including peptides) was extracted by buffer C as described above, then a sonifier was used to assist extraction. Compared to non-ultrasonication, total-protein (including peptides) extracted by the assistance of the sonifier was not significantly increased, as shown in Figure 2. Peptides in effluent were eventually collected after the total-protein extracts were ultrafiltered by a 10 kD ultrafilter. Peptides smaller than 10 kD accounted for 0.72% of total-protein without ultrasonication, compared to 0.84% with ultrasonication, as shown in Figure 2b. 

#### 2.1.3. Peptide Extraction from Protoplasts

**Figure 3:**
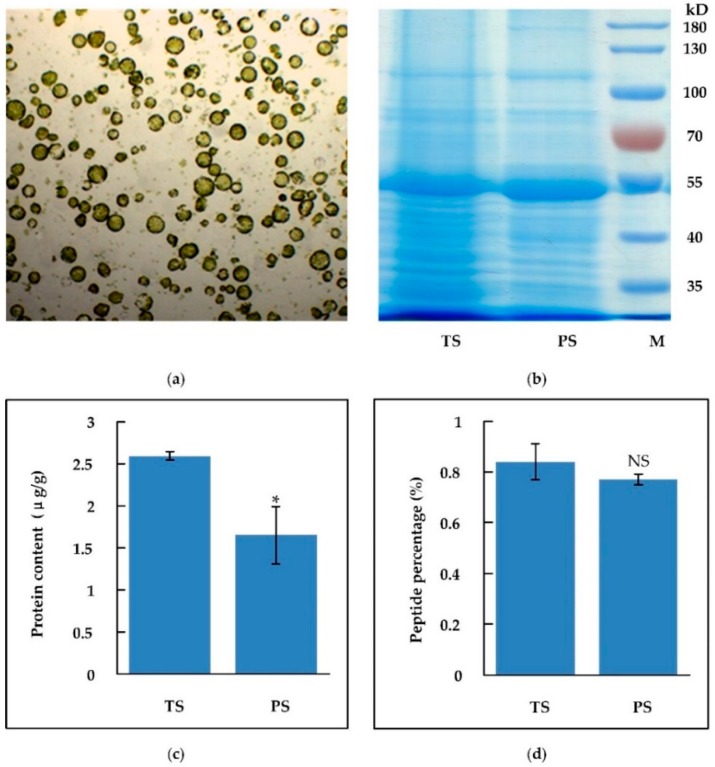
Peptides extracted from leaf tissue and protoplasts. (**a**) Protoplasts of *Arabidopsis* leaf; (**b**) total-protein (including peptides) yield extracted from tissues (TS) and protoplasts (PS); (**c**) total-protein (including peptides) content of tissues (TS) and protoplasts (PS); (**d**) peptide (<10 kD) proportion in total-protein of tissue (TS) and protoplast (PS). Asterisk (*) represents significant difference (*P* < 0.05). NS represents no significant difference. Error bar represents the mean ± standard deviation (n = 3).

Unlike animal cells, plant cells are surrounded with a cell wall consisting of cellulose, pectin, and other components. Traditionally, protein extraction from plant tissue requires mechanical crushing of the cell wall. However, mechanical grinding may give rise to fake peptides derived from the breakup of long peptide chains in larger proteins. Without the protection of the cell wall, protein (including peptides) extraction from plant protoplasts is easier than from plant tissues. Therefore, to avoid the interference by undesirable fragmentization of larger proteins, we extracted protein/peptides by using plant protoplasts instead of plant tissues. Peptides (MW < 10 kD) extracted from leaf tissues by buffer C coupled with ultrasonication (tissue sample, TS) and the one extracted from leaf protoplasts by PBS buffer (protoplast sample, PS) were respectively used for SSP analysis. After digestion with cellulase and pectinase in 8% mannitol, the cell wall was removed, and plant protoplasts were released. The protoplasts in the pellet were collected through low speed centrifuging. When resuspended in PBS buffer, protoplasts would rupture because of imbalanced osmotic pressure and the cellular contents, including proteins and peptides, would leak into the PBS buffer. In our assay, one gram of fresh leaf produced about 4.55 × 10^7^ cells/protoplasts, as shown in Figure 3a. The total-protein yield by protoplast-based extraction was 1.65 μg protein per gram leaf, while peptide content was 0.77%, as shown in Figure 3b–d. Compared to the traditional extraction through frequently-used lysate buffers under ultrasonic assistance, the percentage of peptides was similar between the two extraction methods, although the total-protein content extracted from protoplasts was slightly decreased, as shown in Figure 1, Figure 2 and Figure 3. 

### 2.2. Peptide Identification in Protoplasts

**Figure 4:**
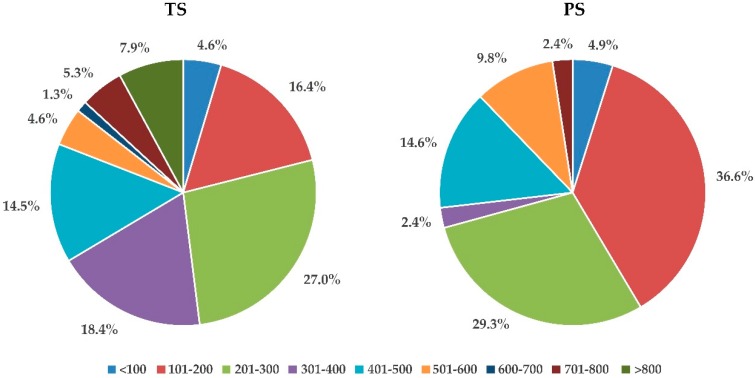
Distribution of precursors according to the peptide chain length (aa) in different samples.

The peptides in TS and PS were identified by a TripleTOF 5600 plus mass spectrometer (AB SCIEX, Foster City, CA, USA) coupled with the Eksigent nanoLC system. The data were analyzed by the ProteinPilot integrated The *Arabidopsis* Information Resource (TAIR) protein database. By using the identified peptides as a “query sequence”, their precursor sequences were searched and downloaded from the TAIR protein database. We examined the length data of the identified peptides and their precursors. Overall, the protoplast-based extraction covered a higher proportion of shorter peptides than traditional extraction. Precursors with less than 200 amino acid residues were 21.05% in TS but 41.46% in PS, as shown in Figure 4. Generally, most SSPs have less than 20 amino acid residues in mature form, and their precursors are also shorter than 200 amino acid residues [5]. Therefore, these results indicated that relatively more potential SSPs could be identified in PS than in TS.

We aligned the sequences of the identified peptides and their precursors by local software “Clastalx”. The identified peptides were categorized according to the homology. The results showed that more homologous peptides belonging to a certain precursor were found in TS than in PS. For example, the maximum number of identified peptides belonging to the same precursor in TS reached 59, while it was merely 16 in PS. Moreover, most identified peptides belonging to a certain precursor in PS were similar fragments with differences of only 1–3 amino acid residues, as shown in Supplemental Materials—Table S1. It was supposed that these peptides were derived from the breakup of peptide chains of larger proteins. This implied that mechanical grinding in traditional extraction may produce more peptide fragments. While in protoplast-based extraction, most precursors owned less than three peptides, accounting for 85.37%, among which 56.1% owned one unique peptide, as shown in Figure 4. In addition, we analyzed the percentage of potential SSPs (peptide chain less than 200 aa) in identified precursors. Obviously, a much higher proportion of potential SSPs was found in PS, implying protoplast-based extraction was helpful in discovering potential SSPs, because its mild conditions could better ensure the protein/peptide integrity, as shown in Figure 4.

Plant protoplast was rarely used as the material for peptide extraction although it has been previously applied in protein expression analysis. Moss (*Physcomitrella patens*) protoplast has been used for identification of peptides [46], but no application of plant protoplast in peptidomics was reported. By using moss protoplast as the material, 323 peptides derived from 79 proteins were identified and the amount of peptides identified in protoplasts was almost six times greater than in the protonemata and five times greater than in gametophores [46]. Our study indicated that extracting protein and peptides from plant protoplast is feasible. Although the yield of total protein extracted from protoplast was a little lower than that extracted from tissue, the peptide production was similar. Taking account of the mild condition to retain protein integrity, extracting peptides from protoplast may be better for plant peptidomic analysis.

### 2.3. Effect of Auxin on Peptidomics

**Figure 5:**
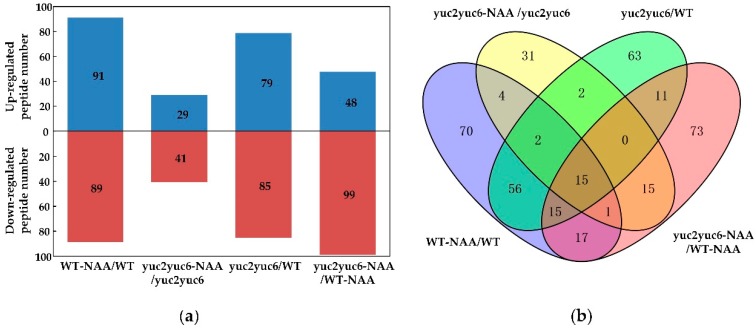
The difference analysis of identified potential SSPs. (**a**) Number of potential SSPs with significant changes. (**b**) Venn diagram of changed potential SSPs among different combinations. WT: wild type.

**Figure 6:**
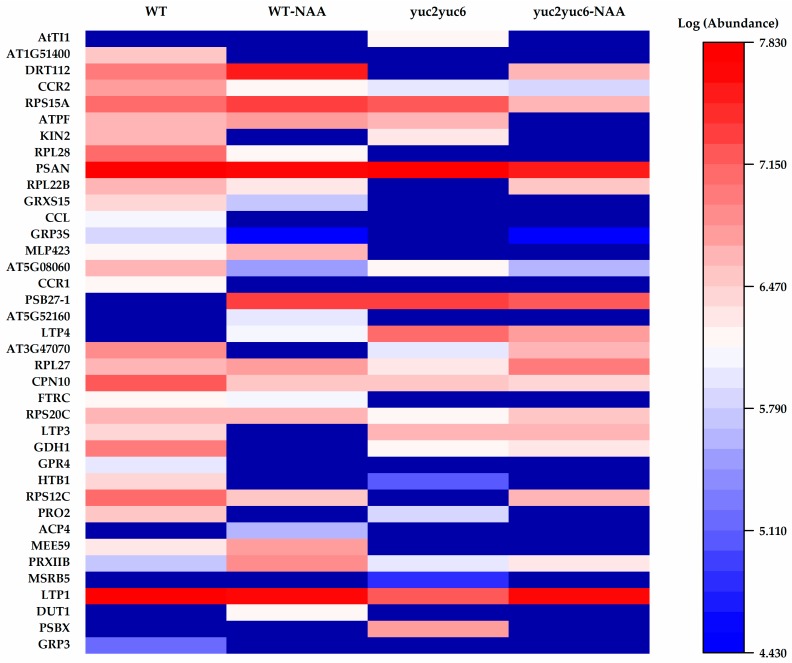
Abundance of potential SSPs in different *Arabidopsis* samples. WT-NAA represents wild type treated with NAA. *yuc2yuc6*-NAA represents *yuc2yuc6* treated with NAA.

To screen the auxin-responsive SSPs, we treated the wild type and auxin biosynthesis defective double mutant *yuc2yuc6* of *Arabidopsis* with a synthetic auxin NAA, and then endogenous peptides identified from leaf protoplasts from the NAA treated and untreated plants were profiled and compared. In this assay, a total of 2665 peptides derived from 416 precursors were identified and quantified through MS/MS. Peptide differences were in compliance with the established fold change criteria (ratio <0.5 or >2). Comparing the abundance of peptides from the protoplasts, we found that 127 small peptides showed significant changes, as shown in Figure 5 and Figure 6. Among them, 60 peptides showed higher abundance in wild type (WT), 7 peptides showed higher abundance in the *yuc2yuc6* mutant, and 13 peptides showed higher abundance in WT with NAA treatment, as shown in Figure 5 and Supplemental Materials—Table S1.

Among these 127 identified peptides, 44 peptides belong to the sequences of ribosomal protein or subunit; 18 peptides are proteases, i.e., reductase, decarboxylase, peroxidase, pyrophosphatase, dismutase, and ATPase; 11 peptides are the fragments of photosystem proteins; 7 peptides are redoxin protein, i.e., cupredoxin, ferredoxin, thioredoxin, and glutaredoxin; and 4 peptides are histone protein. SSPs were defined for the function of cell-to-cell communication and are characterized by cleaved signal peptide sequences. Three classes of identified peptides responding to auxin well meet the above criteria. They were glycine-rich RNA-binding proteins (GRPs), lipid transfer proteins (LTPs), and defensin-like family proteins (DLPs). Five GRPs were identified, i.e., GRP3, GRP4, glycine-rich protein 3 short isoform (GRP3S), cold-circadian rhythm-RNA binding 1 (CCR1), and CCR2. Among the GRPs, GRP3, GRP4, and CCR1 were observed in WT, while GRP3S was absent in the *yuc2yuc6* mutant. CCR2 abundance was much higher in WT than in the *yuc2yuc6* mutant. GRPs were reportedly involved in stress regulation and response to phytohormones, and they were also proven to respond to auxin in our experiment. In addition, abundance of LTP1 showed significant difference between WT and the *yuc2yuc6* mutant. LTP1 was reportedly involved in lipid transport and cell wall organization [47] and was capable of interacting with ethylene [48]. In our experiment, LTP abundance was positively responsive to auxin, implying that LTPs may also take part in auxin signaling. LTP3 was absent in WT treated with NAA and showed higher abundance in the *yuc2yuc6* mutant. LTP4 was absent in WT and was increased in the *yuc2yuc6* mutant with NAA treatment. TRYPSIN INHIBITOR PROTEIN 1 (TI1) and TI3, belonging to DLPs, were respectively observed in WT and *yuc2yuc6*. Being a putative trypsin inhibitor protein functioning in defense against herbivory and fungus, TI1 has been reportedly regulated by brassinosteroids at transcription level [49]. Here, we found that TI1 was regulated by auxin at a protein level. Based on the above peptidomic results, future physiological and molecular studies will be needed to further elucidate the roles and mechanism of these auxin-responsive SSPs.

## 3. Materials and Methods

### 3.1. Reagents/Chemicals and Instrumentation

The urea was purchased from Amresco (Solon, OH, USA). Thiourea was purchased from Pharmabiology. DL-dithiothreitol (DTT), ethylene diamine tetraacetic acid (EDTA), sodium dodecyl sulfate (SDS), and Tris base were purchased from Solarbio (Shanghai, China). Phenylmethanesulfonyl fluoride (PMSF) and protein marker was purchased from Thermo Fisher Scientific (Waltham, MA, USA). Iodoacetamide (IAM) was purchased from Aladdin (Shanghai, China). HPLC-grade acetone, acetonitrile, and formic acid were purchased from TEDIA Company Inc (Fairfield, OH, USA). Cellulose, pectolyase, and macerozyme were purchased from Sigma-Aldrich (St. Louis, MO, USA). Mannitol and other common chemicals and reagents were obtained from the Shanghai Sangon Biotech Company (Shanghai, China). Protein Assay Dye Reagent Concentrate was purchased from Bio-Rad (Hercules, CA, USA). An SDS-PAGE gel preparation kit was purchased from Beyotime (Shanghai, China). Ultrafilter (10 kD) was purchased from Merck-Millipore (Darmstadt, Germany). Milli-Q water was used in all experiments. 

### 3.2. Plant Materials and Growth Conditions

The *Arabidopsis* seeds of wild type and T-DNA insertion mutant *yuc2* (SALK_030199) and *yuc6* (SALK_093708) lines in the Columbia-0 background were ordered from the Arabidopsis Biological Resource Center (https://abrc.osu.edu/). The double mutant *yuc2yuc6* was obtained by crossing *yuc2* with *yuc6*. Seeds of the wild type and mutants were surface-sterilized in 70% ethyl alcohol for 30 s and then placed in 1% sodium hypochlorite solution for 5 min, followed by washing in sterile distilled water 3–5 times. Sterilized seeds were dispersed in 0.1% agar and plated on 1/2 MS medium solidified by 0.8% agar. Seeds were placed at 4 °C for 3 days of vernalization, and then plants were grown at 22 °C under 16 h light/8 h dark. Seven-day old seedlings were transplanted into soil and kept under the same conditions. Three weeks later, 10 μM NAA was drop-irrigated into soil around roots. Leaves were collected for next step protein extraction and protoplast isolation.

### 3.3. Total-Protein Extraction in Plant Leaves

*Arabidopsis* leaves (2 g) were frozen in liquid nitrogen and thoroughly ground to powder with steel beads in a tube by the TissueLyser homogenizer (Qiagen, Germany). The fine powder was mixed with five times in volume of different protein lysate buffers using a vortex. Protein lysate buffers were buffer A (50 mM Tris, 150 mM NaCl, pH 7.5), buffer B (50 mM Tris-HCl, 150 mM NaCl, 1 mM EDTA, 5% glycerol, 1% TritonX-100, pH 7.5), and buffer C (7 M urea, 2 M thiourea, 3.5 mM SDS, 0.01 M Tris, 2 mM EDTA, 10 mM DTT). The final concentration of 1 mM PMSF was added to the homogenates. The total-protein sample in supernatant was pipetted into a new clean tube after centrifuging at 12,000 g for 30 min under 4 °C. 

### 3.4. Protoplast Preparation and Total-Protein Extraction

Compositions of the protoplast lysate buffer were as follows: 1% cellulose R10, 0.1% pectolyase Y23, 0.1% macerozyme R10, 0.4 M mannitol, 0.1 M CaCl_2_, 0.1% Bull Serum Albumin (BSA). The mix was filtrated with a 0.22 μm filter. Leaves (2 g) were cut into filamentous fragments of less than 1 mm and then immerged in protoplast lysate buffer. Leaf fragments were digested in the dark under 25 °C with gentle shaking for 3 to 5 h. The digestion mix was filtrated with a 150 μm nylon fabric filter. The filtrate was centrifuged at 100 g for 5 min and the protoplasts in the pellet were collected. For total-protein extraction, the protoplasts were resuspended in PBS buffer, and the final concentration of 1 mM PMSF was added into the suspension. The total-protein sample in supernatant was collected after centrifuging at 12,000 g for 30 min under 4 °C.

### 3.5. Peptide Sample Preparation

The total-protein in the sample was quantified by the Bradford assay. A defined amount of protein was reduced with 50 mM DTT, followed by incubation in the dark at room temperature for 30 min for modification with 100 mM IAM. The total-protein concentration was determined by Bradford assay and proteins were visualized in SDS-PAGE gel. To obtain peptides, protein extracts were filtered by ultrafilter (10 kD) through centrifugation at 10,000 g for 30 min under 4 °C. The peptides in effluent were collected and desalted with a C18 column. 

### 3.6. Bradford Assay and SDS-PAGE 

A Bradford assay was performed according to the user manual of the Protein Assay Dye Reagent Concentrate. BSA solutions of 0.1 to 1 mg/mL were used as protein standards. A standard solution (800 μL) and 200 μL of dye reagent were mixed with a vortex. The absorbance value at 595 nm was measured after incubation at room temperature for 5 min. Protein/peptide contents were calculated according to the standard curve. SDS-PAGE gel (10%) was prepared following the kit’s instructions. Then, 60 μL of protein sample was mixed with 20 μL of 4× loading buffer. Twenty microliters of mixed sample were injected into the gel for electrophoresis after boiling for 5 min. 

### 3.7. Peptide Analysis through TOF-MS/MS

Samples were analyzed using an AB SCIEX Triple TOF 5600 plus mass spectrometer coupled with the SCIEX Eksigent nanoLC system. The peptide samples were re-dissolved in 2% acetonitrile/0.1% formic acid. Peptide solution was loaded into a C18 trap column (5 μm, 100 μm × 20 mm). The column temperature was maintained at 45 °C. Two solutions including buffer A (2% acetonitrile/0.1% formic acid/98% H_2_O) and buffer B (98% acetonitrile/0.1% formic acid/2% H_2_O) were employed as the mobile phase. Peptides were separated on a C18 analytical column (3 μm, 75 µm × 150 mm). Eluent flow rate was set at 300 nL/min. Column effluent was analyzed by mass spectrometry using high-definition mass spectrometry with the elevated collision energies acquisition method at continuum positive ion resolution MS mode. Source conditions were as follows: “capillary voltage”, 3.0 kV; “source temperature”, 120 °C; “sampling cone”, 60 V; “desolvation temperature”, 350 °C; “cone gas flow”, 50 L/h; “desolvation gas flow”, 500 L/h; “nebulizer gas flow”, 6 bar. For information dependent acquisition, “survey scans” were acquired in 250 ms and production was collected at 50 ms/per scan. MS1 spectra were collected in the range 350–1500 *m/z*, and MS2 spectra were collected in the range of 100–1500 *m/z*. Precursor ions were excluded from reselection for 15 s.

### 3.8. Data Analysis

The original mass spectrometric data were submitted to ProteinLynx ProteinPilot Software v4.5 (AB SCIEX, Foster City, CA, USA) for data processing and analysis. Chromatographic and mass spectrometric peak width resolutions were automatically calculated by the software. The workflow parameters were set as follows: “search type” was MS/MS ion search; “instrument” was TripleTOF 5600 (AB SCIEX, Foster City, CA, USA); “none protein enzyme digestion”; “Cys alkylation” was iodoacetamide; “ture” for bias correction and “background correction” to check protein quantification and normalization; “biological modifications” were selected as ID focus; “search effort” was thorough ID; “protein mass” was set to unrestricted. For protein identification, the “Paragon Algorithm” integrated into “ProteinPilot” and the TAIR protein database was employed for database searching.

### 3.9. Bioinformatics and Annotations

Identified protein sequences were mapped with “Gene Ontology Terms” (http://geneontology.org/) to determine the biological and functional properties of all the identified proteins/peptides. A homology search was performed for all the identified sequences with a localized National Center for Biotechnology Information (NCBI) “blastp” program against the TAIR protein database for “annotation” with an “e-value” setting of less than 1e-5. The “GO term matching” was performed with “blast2go v4.5”. The “Clusters of Orthologous Groups of Proteins System” (COG, http://www.ncbi.nlm.nih.gov/COG/) was employed for the functional annotation of genes from new genomes and for research into genome evolution. To determine whether the identified proteins/peptides are signal proteins/peptides, their sequences were searched with “SignalP5.0” (http://www.cbs.dtu.dk/services/SignalP/). Alignments of proteins/peptides were performed with “Clustalx1.83”.

## 4. Conclusions

Plant SSPs, similar to phytohormones, are vitally important for plant growth and development, as well as in the responses to the changing environment. However, identification technology of mature SSPs is one of the bottleneck limiting factors for SSP studies. In this study, we developed a new method based on protoplast extraction to extract peptides, which was proven to be practical for peptidomic analysis. Under the optimized extraction and purification conditions, we have identified 127 potential auxin-responsive SSPs in *Arabidopsis thaliana* through the peptidomics approach based on tandem mass spectrometry identification. 

## Supplementary Materials

The following are available online, Table S1: Auxin-responsive small peptides identified.
